# Baclofen as a therapeutic option for gastroesophageal reflux disease: A systematic review of clinical trials

**DOI:** 10.3389/fmed.2023.997440

**Published:** 2023-02-17

**Authors:** Erfan Arabpour, Sina Khoshdel, Ali Akhgarzad, Mohammadamin Abdi, Negin Tabatabaie, Dorsa Alijanzadeh, Mohammad Abdehagh

**Affiliations:** ^1^Department of Gastroenterology and Hepatology, Imam Hossein Hospital, Shahid Beheshti University of Medical Sciences, Tehran, Iran; ^2^Department of Internal Medicine, School of Medicine, Shahid Beheshti University of Medical Sciences, Tehran, Iran; ^3^School of Medicine, Shahid Beheshti University of Medical Sciences, Tehran, Iran

**Keywords:** baclofen (PubChem CID: 2284), gastroesophageal reflux disease (GERD), reflux, GABA agonist B, refractory, benign esophageal disease

## Abstract

**Background:**

The main components of gastroesophageal reflux disease (GERD) management include a combination of medications and lifestyle modifications; Nevertheless, based on the severity of symptoms and their response to medications, other treatments could be considered. Baclofen has been demonstrated in studies to relieve GERD symptoms. The current study aimed to precisely address the effects of baclofen on the treatment of GERD and its characteristics.

**Methods:**

A systematic search was carried out in Pubmed/Medline, Cochrane CENTRAL, Scopus, Google Scholar, Web of Science, and clinicaltrials.gov up to December 10, 2021. The search terms included baclofen, GABA agonists, GERD, and reflux.

**Results:**

We selected 26 papers that matched the inclusion criteria after examining 727 records. Studies were classified into four categories based on the study population and reported outcomes: (1) adults, (2) children, (3) patients with gastroesophageal reflux-induced chronic cough, (4) hiatal hernia patients. The results revealed that baclofen can significantly improve reflux symptoms and pH-monitoring and manometry findings to different degrees in all four mentioned categories; although its effect on pH-monitoring parameters seems less significant than the other parameters. Mild neurological and mental status deterioration were the most reported side effects. However, side effects occurred in a portion of less than 5% of short-term users and nearly 20% of long-term users.

**Conclusion:**

In PPI-resistant patients, a trial of adding baclofen to the PPI may be helpful. Baclofen therapies may be more beneficial for symptomatic GERD patients who also report concurrent conditions including alcohol use disorder, non-acid reflux, or obesity.

**Systematic review registration:**

https://clinicaltrials.gov/.

## 1. Introduction

Gastroesophageal reflux disease (GERD) is a clinical condition caused by the chronic retrograde reflux of acidic contents of the stomach into the esophagus with discomforting symptoms or complications or both (1). Gastroesophageal reflux (GER) is a physiological condition during infancy and childhood and may not require treatment. In children, GERD is the symptomatic reflux of gastric contents into the esophagus and should be treated according to the severity of symptoms (2, 3). Global surveys in 2017 estimated an 18.1% increase in the total prevalence of GERD cases and a 67.1% increase in the years lived with disability (YLD) compared to 1990 (4). These findings suggest GERD as a public health concern with a considerable socioeconomic burden in the near future.

Diagnosis of GERD is based on clinical symptoms (heartburn, regurgitation, and non-cardiac chest pain) and response to empiric proton pump inhibitors (PPIs). Nonetheless, studies have shown limitations of non-objective diagnosis; as a result, diagnostic evaluation such as upper endoscopy is recommended based on the clinical setting, especially in patients with red flags like dysphagia (5).

The main components of GERD management are lifestyle modification and PPIs. Nevertheless, based on the severity of symptoms and their response to PPIs, H2blockers, baclofen, antacids, sucralfate, prokinetic agents, and invasive anti-reflux procedures (such as surgeries, sphincter augmentation, and endoscopic therapy) are considered in combination with other treatments or as the replacement therapy for patients. Consideration is required based on efficacy and tolerability profile of each treatment option (5–7).

The pathophysiology of GERD is multifactorial and is explained by natural anti-reflux barrier. Studies suggest following mechanisms for GERD: the hypotonic lower esophageal sphincter (LES), hiatal hernia, Gubaroff valve failure, and thoraco-abdominal pressure (8, 9). Also, studies have shown that baclofen has been highly beneficial in the treatment of refractory GERD. Baclofen is an FDA-approved agonist of the gamma-aminobutyric acid (GABA) receptor, which is generally used for the relaxation of pathologic spasms originating from the central nervous system (CNS). Its mechanism of action on GERD is by inhibition of LES relaxation induced *via* vasovagal reflexes. Baclofen inhibits these reflexes through GABA_*B*_ receptor activation (10, 11).

Although previous studies proved effectiveness of baclofen on GERD (12), to the date, there are no systematic reviews regarding outcomes and side effects in different patient (grouped by: age, and comorbidities); A systematic review in that regard might resolve probable hesitancies in prescribing baclofen. Therefore, we will conduct this systematic review, aiming to facilitate informed decisions in managing GERD using baclofen. To achieve this goal we will review efficacy, side effects, and response predictors in different patients grouped by: age (adult vs. pediatrics), comorbidities (hiatal hernia and GERD-related chronic cough), and some other factors.

## 2. Materials and methods

This study was conducted and reported according to the Preferred Reported Items for Systematic Reviews and Meta-Analysis (PRISMA) statement (13).

### 2.1. Search strategy

We searched Pubmed/Medline, Cochrane CENTRAL, Scopus, Google Scholar, Web of Science, and Clinicaltrials.gov for studies reporting the efficacy/effectiveness of baclofen in patients with GERD, published up to December 10, 2021. The search terms were baclofen, GABA agonists, GERD, and reflux. No language restrictions were imposed.

### 2.2. Study selection

The records found through database searching were merged, and the duplicates were removed using EndNote X9. Two authors independently screened the records by title/abstract and full-texts to exclude those unrelated to the study topic. Included studies met the following inclusion criteria: (i) patients were diagnosed with GERD based on a defined criterion; (ii) patients were treated with baclofen; and (iii) treatment outcomes were recorded. Conference abstracts, reviews, experimental studies on animal models, and articles that their full-text or original data were not available were excluded.

### 2.3. Data extraction

Two authors designed a data extraction form. These reviewers extracted the following items from all eligible studies: first author’s name, year of publication, country/ies where the research was conducted, type of epidemiological study, demographics, treatment protocols, adverse effects, and outcomes. Data was inserted into an excel sheet, and differences were resolved by consensus.

### 2.4. Quality assessment

The checklists provided by the National Institute of Health (NIH) for controlled intervention and before-after (pre-post) studies with no control group were used to perform the quality assessment (14).

## 3. Results

**FIGURE 1:**
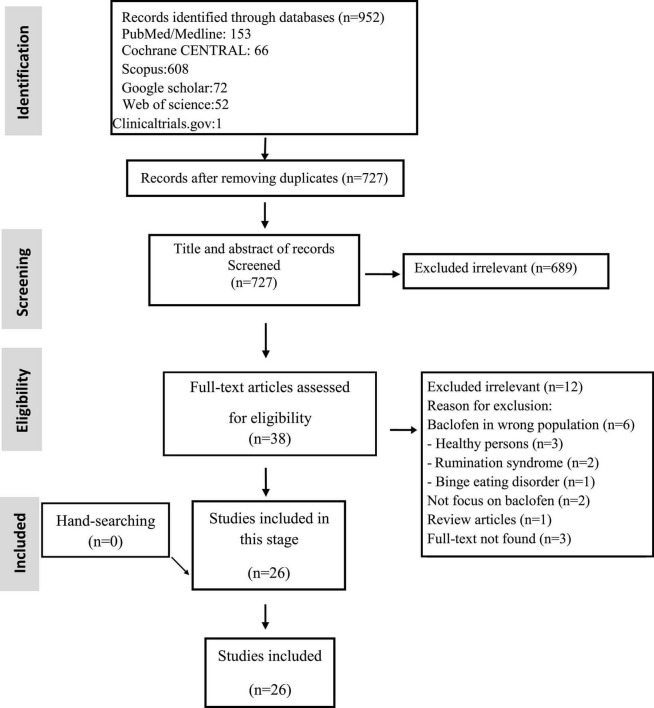
Flow chart of study selection for inclusion in the systematic review and meta-analysis.

We investigated a total of 952 records found in the systematic search; after removing duplicates and full-text reviews, 26 were chosen. Studies included and excluded through the review process are summarized in Figure 1 and Supplementary Table 1. Among the included studies, there were 9 crossover RCTs, 8 RCTs, and 9 single-arm clinical trials. The studies originated from twelve countries: United States (*n* = 6), China (*n* = 4), Iran (*n* = 3), Australia, Belgium, Italy, Sweden (*n* = 2, for each one), Switzerland, the Netherlands, Mexico, Japan, and Germany (*n* = 1, for each one) (Table 1). Two of the studies had two separate parts (15, 16), so we looked at these parts separately and overviewed 28 studies as a whole. All studies assessed baclofen efficacy based on clinical status, pH monitoring, or manometry findings. Additionally, in most trials, the safety of treatment was evaluated by the occurrence of adverse events or side effects.

**TABLE 1:** Characteristics of included studies.

References	Country	Study design	Purpose
Curcic et al. (28)	Switzerland	RCT, crossover	Effects of baclofen on the functional anatomy of the OGJ and proximal stomach in adult GERD patients
Abbasinazari et al. (18)	Iran	RCT	Effect of co-administration of omeprazole plus baclofen compared to omeprazole plus placebo on alleviation of symptoms in adult patients with GERD
Ciccaglione et al. (16)	Italy	RCT	Effects of acute and chronic administration of baclofen on 24 h esophageal and gastric pH patterns in adult patients with GERD
Beaumont et al. (15)	Netherlands	RCT, crossover	Effect of baclofen during PPI treatment on gastroesophageal reflux in GERD patients with no hiatal hernia compared to those with a large hiatal hernia
Cange et al. (31)	Sweden	RCT, crossover	Effect of baclofen on esophageal acid exposure in adult patients with GERD
Cossentino et al. (25)	USA	RCT	Effect and tolerability of baclofen in adult GERD patients over 2 weeks
Dibner et al. (17)	Mexico	RCT	Effect of baclofen on TLESR, gastroesophageal reflux and gastric emptying in children with GERD
Gerson et al. (54)	USA	RCT, crossover	Efficacy and safety of Arbaclofen Placarbil for decreasing meal-induced reflux episodes in adult patients with GERD
Grossi et al. (32)	Italy	RCT	Effect of baclofen on 24-h esophageal and LES motility in a group of GERD patients after multiple oral doses of the drug
Omari et al. (35)	Australia	RCT	Effect of baclofen on the rates of TLESR, gastroesophageal reflux, and gastric emptying in children with GERD.
van Herwaarden et al. (34)	Sweden	RCT, crossover	Effect of baclofen on reflux symptoms, esophageal pH and lower esophageal sphincter manometry in GERD patients.
Vela et al. (27)	USA	RCT, crossover	Compare the frequencies of postprandial GER and associated symptoms after treatment with placebo and baclofen in heartburn patients
Scarpellini et al. (33)	Belgium	RCT, crossover	Investigate the effect of baclofen on the presence and extension of an acid pocket in naive GERD patients with heartburn as the predominant symptom
Zhang et al. (11)	Australia	RCT, crossover	Effect of baclofen on TLESRs and postprandial gastro-esophageal reflux in patients with reflux disease.
Orr et al. (30)	USA	RCT, crossover	To determine if baclofen would significantly reduce reflux during sleep, and also improve objective and subjective measures of sleep
Sobhani Shahmirzadi et al. (21)	Iran	RCT	Effect of baclofen in pediatric GERD
Vakil et al. (26)	USA	RCT	Efficacy and safety of arbaclofen placarbil over 4 weeks in symptomatic GERD patients
Vadlamudi et al. (55)	USA	Single-arm clinical trial	Evaluate the efficacy of baclofen on symptoms in children with refractory GERD
Xu et al. (56)	China	Single-arm clinical trial	efficacy of baclofen in treating patients with refractory chronic cough induced by gastroesophageal reflux resistant to PPIs
Xu et al. (23)	China	Single-arm clinical trial	Efficacy and safety of baclofen for the treatment of refractory gastroesophageal reflux-induced chronic cough unresponsive to standard anti-reflux therapy
Xu et al. (22)	China	Single-arm clinical trial	Efficacy of baclofen for treating refractory gastroesophageal reflux-induced chronic cough unresponsive to omeprazole and ranitidine
Zhu et al. (42)	China	Single-arm clinical trial	Baclofen effect on pressure and length of the lower esophageal sphincter as predictive indicators of therapeutic efficacy for refractory gastroesophageal reflux-induced chronic cough
Kawai et al. (24)	Japan	Single-arm clinical trial	Effects of baclofen on GERD in neurologically impaired children
Khodadad et al. (29)	Iran	Single-arm clinical trial	Efficacy of baclofen on lower esophageal sphincter in infants
Koek et al. (20)	Belgium	Single-arm clinical trial	Effect of baclofen in patients with persistent non-acid duodenal reflux during PPI therapy
Bajbouj et al. (19)	Germany	Single-arm clinical trial	Influence of an anti-reflux therapy with 80 mg esomeprazole plus baclofen for the treatment of refractory GERD in adult patients

GERD, gastroesophageal reflux disease; LES, lower esophageal sphincter; OGJ, oesophagogastric junction; PPI, proton pump inhibitor; RCT, randomized controlled trial; TLESR, transient lower esophageal sphincter relaxation.

### 3.1. Quality of included studies

Based on the NIH checklists for controlled intervention and before-after (pre-post) studies with no control group, the included studies had a low risk of bias (Supplementary Tables 2, 3).

### 3.2. Patient characteristics

Except for one study that did not report the number of patients (17), the remaining 27 trials included 785 patients who got baclofen and 358 who received control medication. The baclofen groups included individuals aged 7.1 months (infants) to 58 years (adults). The most frequently utilized methods for diagnosing GERD were, in order, history, pH monitoring, manometry, and endoscopy (Table 2).

**TABLE 2:** Patients characteristics.

References	Study design	Case definition	Control definition	Case population (M/F)	Control population (M/F)	Age (case/control) (yr)	Way of reflux diagnosis
Curcic et al. (28)	RCT, crossover	GERD patients with erosive esophagitis or pathological esophageal acid exposure who received baclofen	GERD patients with erosive esophagitis or pathological esophageal acid exposure who received placebo	12 (7/5)	12 (7/5)	37 (37/37)	Endoscopy based on LA-classification and/or pathological findings on pH monitoring
Abbasinazari et al. (18)	RCT	Patients with a diagnosis of GERD who received baclofen in addition to omeprazole as treatment	Patients with a diagnosis of GERD who received placebo in addition to omeprazole as treatment	25 (11/14)	28 (13/15)	NR(41.0/36.8)	History (mayo gastroesophageal reflux questionnaire)
Ciccaglione et al. (16) (1)	RCT	Patients chose based on clinical symptoms indicating GERD for at least 3 months before enrollment who received baclofen for 1 day	Patients chose based on clinical symptoms indicating GERD for at least 3 months before enrollment who received placebo for 1 day	15	13	40	History (clinical symptoms)
Ciccaglione et al. (16) (2)	RCT	Patients chose based on clinical symptoms indicating GERD for at least 3 months before enrollment who received baclofen for 4 weeks	Patients chose based on clinical symptoms indicating GERD for at least 3 months before enrollment who received placebo for 4 weeks	10	6	45	History (clinical symptoms)
Beaumont et al. (15) (1)	RCT, crossover	GERD patients with no hiatal hernia taking a PPI for at least 3 months before the study who received baclofen (treatment with PPI was continued)	GERD patients with no hiatal hernia taking a PPI for at least 3 months before the study who received placebo (treatment with PPI was continued)	16 (8/8)	16 (8/8)	54	History
Beaumont et al. (15) (2)	RCT, crossover	Patients with a large hiatal hernia (≥3 cm) taking a PPI for at least 3 months before the study who received baclofen (treatment with PPI was continued)	Patients with a large hiatal hernia (≥3 cm) taking a PPI for at least 3 months before the study who received baclofen (treatment with PPI was continued)	11 (7/4)	11 (7/4)	58	History
Cange et al. (31)	RCT, crossover	GERD patients with a history of esophagitis and/or time of esophageal acid exposure (pH < 4) of more than 5% on 24-h pH monitoring who received baclofen	GERD patients with a history of esophagitis and/or time of esophageal acid exposure (pH < 4) of more than 5% on 24-h pH monitoring who received placebo	20 (15/5)	20 (15/5)	41.2 (41.2/41.2)	History, Los Angeles classification grade A–C or pH monitoring
Cossentino et al. (25)	RCT	Symptomatic GERD patients with the evidence of upright or supine reflux on 24-h pH testing who received baclofen	Symptomatic GERD patients with the evidence of upright or supine reflux on 24-h pH testing who received placebo	23 (17/6)	20 (10/10)	49 (47.2/50.3)	History (questionnaires to assess GERD symptoms), esophageal manometry, and 24-h pH monitoring
Dibner et al. (17)	RCT	Neurologically healthy children with GERD with failure of conventional treatment who received baclofen	Neurologically healthy children with GERD with failure of conventional treatment who received placebo	NR	NR	NR(range: 2.6 to 17.4 yr)	NR
Gerson et al. (54)	RCT, crossover	Patients with reported GERD symptoms occurring at least 3 times a week and 20 reflux episodes on impedance-pH monitoring over a period of 2 h who received baclofen	Patients with reported GERD symptoms occurring at least 3 times a week and 20 reflux episodes on impedance-pH monitoring over a period of 2 h who received placebo	44 (22/22)	44 (22/22)	41 (41/41)	History (clinical symptoms) and pH monitoring
Grossi et al. (32)	RCT	Symptomatic GERD patients who received baclofen	Symptomatic GERD patients who received placebo	14	7	43	Endoscopy and manometric exam
Omari et al. (35)	RCT	Children with severe GERD who had failed to improve after routine therapeutic measures that received baclofen	Children with severe GERD who had failed to improve after routine therapeutic measures that received placebo	15 (9/6)	15 (8/7)	10.0 (9.1/11.0)	History (clinical symptoms)
van Herwaarden et al. (34)	RCT, crossover	Symptomatic GERD patients who received baclofen	Symptomatic GERD patients who received placebo	20 (12/8)	20 (12/8)	45.1	History (clinical symptoms)
Vela et al. (27)	RCT, crossover	Heartburn patients receiving baclofen	Heartburn patients who received placebo	9(6/3)	9(6/3)	36 (36/36)	History (clinical symptoms)
Scarpellini et al. (33)	RCT, crossover	Adult GERD patients with heartburn as predominant symptom who received baclofen as treatment	Adult GERD patients with heartburn as predominant symptom who received placebo	13 (6/7)	13 (6/7)	29.8	History (questionnaires)
Zhang et al. (11)	RCT, crossover	Symptomatic GERD patients with evidence of esophagitis on endoscopy who received baclofen	Symptomatic GERD patients with evidence of esophagitis on endoscopy who received placebo	20(15/5)	20(15/5)	56.5 (56.5/56.5)	History (clinical symptoms) and endoscopy (hetzel grading)
Orr et al. (30)	RCT, crossover	Individuals with complaints of nighttime heartburn or regurgitation at least twice per week and a carlsson GERD score of at least 5 who received baclofen	Individuals with complaints of nighttime heartburn or regurgitation at least twice per week and a carlsson GERD score of at least 5 who received placebo	21(9/12)	21(9/12)	43 (43/43)	History (carlsson GERD questionnaire)
Sobhani Shahmirzadi et al. (21)	RCT	6 months to 12 years old children with GERD who received baclofen + PPI	6 months to 12 years old children with GERD who received PPI	54(27/27)	58(20/38)	6.61	NR
Vakil et al. (26)	RCT	Patients with heartburn and/or regurgitation ≥3 days a week and receiving arbaclofen placarbil	patients with heartburn and/or regurgitation ≥3 days a week who received placebo	125(56/69)	31(15/16)	41.8 (41.8/41.8)	History (clinical symptoms)
Vadlamudi et al. (55)	Single-arm clinical trial	Children ages 1 to 18 years with a known diagnosis of GERD receiving baclofen	NA	53(34/19)	NA	6.1	NR
Xu et al. (56)	Single-arm clinical trial	Patients with GERC resistance to proton pump inhibitors who received baclofen	NA	26-year-old male/42-year-old male/63-year-old female	NA	46.3	History (clinical symptoms) and pH monitoring
Xu et al. (23)	Single-arm clinical trial	Patients with suspected refractory GERC who received baclofen	NA	16(9/7)	NA	47.8	History (clinical symptoms) and pH monitoring
Xu et al. (22)	Single-arm clinical trial	Patients with suspected refractory GERC unresponsive to omeprazole and ranitidine who received baclofen	NA	57	NA	NR	History (clinical symptoms) and pH monitoring
Zhu et al. (42)	Single-arm clinical trial	Patients with suspected refractory GERC unresponsive to standard anti-reflux therapy who received baclofen	NA	138(66/72)	NA	51.4	Esophageal manometry and multichannel intraluminal impedance-pH monitoring
Kawai et al. (24)	Single-arm clinical trial	Neurologically impaired children with GERD who received baclofen	NA	8	NA	3	24-h esophageal pH monitoring
Khodadad et al. (29)	Single-arm clinical trial	Infants with GERD (diagnosed by specific clinical criteria) for at least 1 month who received baclofen.	NA	30(17/13)	NA	7.1 months	History (clinical symptoms)
Koek et al. (20)	Single-arm clinical trial	GERD patients with persistent heartburn or regurgitation that treated for at least 3 months with omeprazole 20 mg twice daily who received baclofen.	NA	16 (5/11)	NA	46.2	History (standardized questionnaire for symptoms), endoscopy, and esophageal pH monitoring
Bajbouj et al. (19)	Single-arm clinical trial	Adult GERD patients with persistent pathological pH/MII results despite receiving esomeprazole who were treated with baclofen + esomeprazol	NA	7	NA	NR	History (standardized questionnaire for symptoms) and pH monitoring

GERC, gastroesophageal reflux-induced chronic cough; GERD, gastroesophageal reflux disease; LA, Los Angeles; NA, not applicable; NR, not reported; PPI, proton pump inhibitor; RCT, randomized controlled trial.

### 3.3. Intervention characteristics

In twenty-two studies, the treatment regimen consisted only of baclofen, whereas in the six remaining studies, the treatment regimen consisted of baclofen and a PPI (18–23). The majority of studies used baclofen for at least 1 week; however, some used shorter treatment periods to investigate baclofen’s acute effects. In all studies, treatments were administered orally, except one that also administered enteric baclofen (24) (Table 3).

**TABLE 3:** Intervention characteristics.

References	Case treatment regimen	Treatment duration	Route of delivery
Curcic et al. (28)	Single dose baclofen (400 mg suspension)	Single dose (7 days washout time)	Oral
Abbasinazari et al. (18)	Sustained release baclofen 10 mg tablet BD with omeprazole 20 mg OD	2 weeks	Oral
Ciccaglione et al. (16) (1)	Baclofen 10 mg QID	1 day	Oral
Ciccaglione et al. (16) (2)	Baclofen 10 mg TDS for the first week, and then 10 mg QID for the next 3 weeks	4 weeks	Oral
Beaumont et al. (15) (1)	Baclofen 5 mg TDS, gradually reached 20 mg TDS after 10 days	12 days (at least 7 days washout time)	Oral
Beaumont et al. (15) (2)	Baclofen 5 mg TDS, gradually reached 20 mg TDS after 10 days	12 days (at least 7 days washout time)	Oral
Cange et al. (31)	Baclofen 40 mg single dose	Single dose (at least 4 weeks washout time)	Oral
Cossentino et al. (25)	Baclofen 10 mg TDS, gradually reached 20 mg TDS after 6 days	2 weeks	Oral
Dibner et al. (17)	Baclofen 0.5 mg/kg	Single dose	Oral
Gerson et al. (54)	Baclofen 10, 20, 40, or 60 mg single dose	Single dose (3–7 days washout time)	Oral
Grossi et al. (32)	Baclofen 10 mg QID	1 day	Oral
Omari et al. (35)	0.5 mg/kg baclofen (up to a maximum of 40 mg) single dose	Single dose	Oral
van Herwaarden et al. (34)	Baclofen 40 mg single dose	Single dose (3–10 days washout time)	Oral
Vela et al. (27)	Baclofen 40 mg single dose	Single dose (2–7 days washout time)	Oral
Scarpellini et al. (33)	Baclofen 40 mg single dose	Single dose (1 week washout time)	Oral
Zhang et al. (11)	Baclofen 40 mg TDS	1 day (1 week washout time)	Oral
Orr et al. (30)	Baclofen 40 mg OD	2 days (1 week washout time)	NR
Sobhani Shahmirzadi et al. (21)	Baclofen 0.25 mg/kg divided into two daily doses and omeprazole 1mg/kg	1 month	Oral
Vakil et al. (26)	Baclofen 20, 40, or 60 mg once daily; or 30 mg twice daily	4 weeks	Oral
Vadlamudi et al. (55)	Baclofen 0.5 mg/kg divided into 3 daily doses (maximum daily dose was 30 mg/day)	NR	Oral
Xu et al. (56)	Baclofen 20 mg TDS	8 weeks	Oral
Xu et al. (23)	Baclofen 20 mg TDS + omeprazole 20 mg BD	8 weeks	Oral
Xu et al. (22)	Baclofen 20 mg TDS + omeprazole 20 mg BD	10.96 weeks (median time)	Oral
Zhu et al. (42)	Baclofen 10 mg TDS, gradually reached 20 mg TDS after 9 days	NR	Oral
Kawai et al. (24)	Baclofen 0.7 mg/kg/day in 3 divided doses	7 days	Enteral formula *via* NGT for 7, oral for 1
Khodadad et al. (29)	0.25 mg/kg/day baclofen in 2 divided doses	3 months	Oral
Koek et al. (20)	Baclofen 5 mg TDS, gradually reached 20 mg TDS after 10 days + omeprazole 20 mg BD	14 days	Oral
Bajbouj et al. (19)	Baclofen 5 mg TDS, gradually reached 20 mg TDS after 4 weeks + esomeprazole 40 mg BD	3 months	Oral

BD, twice a day; NGT, nasogastric tube; NR, not reported; OD, once daily; QID, four times a day; RCT, randomized controlled trial; TDS, three times a day.

### 3.4. Outcomes

#### 3.4.1. Safety and side effects

Among 28 studies, four studies did not investigate treatment side effects (17, 25–27). No treatment-related severe adverse events were observed in the remaining 24 studies. The adverse effects noted were somnolence among 14.2% of participants, dizziness (10.0%), fatigue (4.9%), nausea (1.6%), gastrointestinal symptoms (0.9%), headache (0.7%), anxiety (0.2%), and a slight reduction in muscular tone (0.2%). The most frequently reported adverse effects were neurological and mental status deterioration (particularly dizziness and somnolence). However, some of these events were not induced by baclofen; rather, they were caused by the other underlying comorbidities. The side effects occurred in a portion of less than 5% of short-term users (less than 4 weeks) and nearly 20% of long-term users (more than 4 weeks), and also occurred during placebo therapy in certain studies. Baclofen had no adverse effects in seven studies, and was well tolerated (11, 16, 18, 21, 28–30) (Table 4).

**TABLE 4:** Treatment safety and side effects.

References	Treatment side effects
Curcic et al. (28)	None
Abbasinazari et al. (18)	None
Ciccaglione et al. (16) (1)	None
Ciccaglione et al. (16) (2)	2/12 patients withdrew from the study after 10 days of treatment with baclofen, one because of nocturnal anxiety with sleepiness, and the other due to low blood pressure with dizziness. Baclofen was well tolerated in all other patients.
Beaumont et al. (15) (1 and 2)	Somnolence (6/27), dizziness (3/27), and nausea (2/27) occurred with baclofen. One of 27 patients withdrew from the study prematurely because of dizziness. No AEs occurred during the placebo.
Cange et al. (31)	Tiredness and/or mild vertigo (8/20), headache (1/20), GI symptoms (1/20) occurred with baclofen. tiredness and/or mild vertigo (1/20), headache (3/20), GI symptoms (2/20) occurred with placebo.
Cossentino et al. (25)	NR
Dibner et al. (17)	NR
Gerson et al. (54)	Abdominal pain (1/44), constipation (0), nausea (0), diarrhea (1/44), vomiting (0/44), somnolence (1/44), fatigue (1/44), headache (2/44) occurred with baclofen. Abdominal pain (1/44), constipation (2/44), nausea (3/44), Diarrhea (1/44), vomiting (2/44), somnolence (1/44), fatigue (1/44), and headache (3/44) occurred with placebo.
Grossi et al. (32)	Headache (1/14) and dizziness (1/14) occurred with baclofen. No AEs occurred with placebo
Omari et al. (35)	Breathlessness (*n* = 2; 1 placebo group, 1 baclofen group); tiredness (*n* = 2; 1 placebo group, 1 baclofen group) and nausea (*n* = 1; baclofen group). none of these events was considered significant. These symptoms were judged to most likely be reflux disease itself or discomfort caused by the invasive GI procedures rather than baclofen
van Herwaarden et al. (34)	80% of baclofen and 35% of the placebo group experienced mental/neurological AEs (*P* = 0.00001). Other AEs occurred in both groups but were statistically not significant
Vela et al. (27)	NR
Scarpellini et al. (33)	9/13 patients reported mild dizziness and sleepiness with baclofen
Zhang et al. (11)	None
Orr et al. (30)	None
Sobhani Shahmirzadi et al. (21)	None
Vakil et al. (26)	NR
Vadlamudi et al. (55)	3/53 patients withdrew from the study because of side effects. Dose related drowsiness reported from 4 of 50 remained patients
Xu et al. (56)	(1/3) slight dizziness and (1/3) sleepiness occurred with baclofen
Xu et al. (23)	Somnolence (5/16), dizziness(2/16), fatigue (3/16), nausea (1/16), diarrhea (1/16) occurred with baclofen
Xu et al. (22)	Somnolence (*n* = 21/57), dizziness (*n* = 7/57), and drowsiness (*n* = 12/57) occurred with baclofen
Zhu et al. (42)	Somnolence (*n* = 49/138), dizziness (*n* = 33/138), fatigue (*n* = 24/138), nausea (*n* = 4/138), and diarrhea (*n* = 1/138) occurred with baclofen
Kawai et al. (24)	A slight reduction in muscle tone in 1 of 8 patients
Khodadad et al. (29)	None
Koek et al. (20)	Transient nausea in 2 and drowsiness in 3 of 16 patients
Bajbouj et al. (19)	2/7 patients had to discontinue study because of drowsiness

AE, adverse event; GI, gastrointestinal; NR, not reported.

#### 3.4.2. Efficacy

Studies were classified into four categories based on the study population and reported outcomes: (1) adults, (2) children, (3) patients with gastroesophageal reflux-induced chronic cough, and (4) hiatal hernia patients.

##### 3.4.2.1. Outcomes in adults

This category contained seventeen studies. These publications evaluated baclofen’s efficacy using changes in clinical status, acid reflux time, TLESR incidence, GER incidence, LES pressure, and some other parameters.

Eleven trials reported changes in clinical status. Baclofen significantly improved clinical symptoms in seven studies. However, Bajbouj et al. (19) and Zhang et al. (11) discovered no significant improvement following baclofen treatment. According to Abbasinazari et al. (18) baclofen alleviated esophageal symptoms (heartburn and regurgitation) but had no significant effect on extra-esophageal symptoms (chest pain and hoarseness). Baclofen reduced belching with no effect on other reflux symptoms in Cange et al.’s (31) trial.

Changes in acid reflux time were documented in eleven studies. Baclofen significantly decreased acid reflux time in five studies. However, four studies found that baclofen has no significant effect on acid reflux time (11, 15, 19, 20). Orr et al. (30) reported that baclofen reduced recumbent acid contact time by 50% compared to placebo, although the difference was not statistically significant. In Curcic et al.’s (28) study, baclofen increased reflux duration by a small but significant degree.

Four studies reported changes in TLESR incidence. All of them mentioned that baclofen has a significant reducing effect on TLESR incidence (11, 32–34).

Thirteen studies reported changes in GER incidence. All of them declared baclofen had a significant effect on reducing GER incidence, except for two studies: (a) Koek et al. (20) reported that after adding baclofen, the number of acid reflux episodes remained unchanged, but the number of duodenal reflux episodes and the number of duodenal reflux episodes lasting longer than 5 min decreased significantly. (b) Bajbouj et al. (19) observed no significant changes in reflux episodes, before and after adding baclofen. Additionally, two trials examined the effect of body posture on efficacy of baclofen (15, 25). Both of them suggested that baclofen decreased reflux episodes in the upright position significantly, while reflux episodes in the supine position did not change significantly.

Six studies investigated changes in LES pressure. Baclofen significantly elevated LES pressure in four studies (11, 28, 32, 33). Cossentino et al. (25) and van Herwaarden et al. (34) on the other hand, found no difference in LES pressure between the baclofen and placebo groups. All outcomes are present in more detail in Table 5.

**TABLE 5:** Outcomes in adults.

References	Baclofen on					
	Clinical status	Acid reflux time	TLESR incidence	GER incidence	LES pressure	Others
Curcic et al. (28)	NR	Baclofen increased reflux duration in patients with GERD by a small but significant degree (*P* < 0.0001)	NR	Baclofen reduced the number of reflux events from 3 to 2 in GERD patients resulting in a 40% reduction (*P* < 0.0001)	Treatment increased LES pressure in patients with GERD by 4.50 ± 1.49 mmHg (*P* < 0.003) and intra-abdominal LES length by 0.35 ± 0.06 cm (*P* < 0.0001)	Gastric emptying was faster during baclofen treatment; nevertheless, measurement variability was high and this change was not statistically significance. Baclofen reduced the esophagogastric insertion in GERD patients by 4.09° ± 1.82° (*P* = 0.025). Baclofen had no effect on the change in proximal gastric curvature
Abbasinazari et al. (18)	Significant differences were observed between the two groups in the prevalence of heartburn (*p* < 0.0001) and regurgitation (*p* < 0.0001); baclofen had no significant effect on chest pain (*p* = 0.35) or hoarseness (*p* = 0.93) compared to placebo	NR	NR	NR	NR	The total GERD score (the sum of the scores for all four symptoms; heartburn, acid regurgitation, chest pain, and hoarseness) was significantly affected by baclofen (*p* < 0.0001)
Ciccaglione et al. (16) (1)	NR	In baclofen group, there was a highly significant reduction in percent time with pH < 4 (–57.81%). During placebo, no significant change was observed for percent time pH < 4 (*p* = NS)	NR	In all GERD patients, baclofen reduced the number of reflux episodes (–51.01%). During placebo, there was no significant change in the number of reflux episodes (*p* = NS). In the placebo group, patients had a statistically significant higher average number of reflux episodes compared to the patients in the baclofen group (*p* < 0.003).	NR	A statistically significant increase in mean gastric pH value in the 24-h period was observed 15 GERD patients who received baclofen (*p* < 0.0004). No change was observed in the placebo (*p* = NS).
Ciccaglione et al. (16) (2)	The intensity and frequency of symptoms were significantly improved in patients after receiving baclofen, while in the placebo group the total symptom scores was not changed. The number of antacid tablets consumed per week was 7 before placebo, 8 during placebo (NS), 8 before baclofen, and 2 during baclofen (*p* < 0.01).	The percent time pH < 4 was significantly decreased after the treatment with baclofen compared to the values reported at the beginning of the treatment (–53.45%). The percent time with pH < 4 in GERD patients treated with placebo was not significantly changed (*p* = NS)	NR	The number of reflux episodes was significantly decreased after the treatment with baclofen compared to the values reported at the beginning of the treatment (–76.36%). The median number of reflux episodes in GERD patients receiving placebo was not significantly changed (*p* = NS)	NR	The number of reflux episodes longer than 5 min was assessed in five patients treated with baclofen and in three patients treated with placebo. In baclofen group, a significant reduction was noted (*p* < 0.002) while no change was observed in placebo group (*p* = NS).
Beaumont et al. (15) (1)	NR	The total acid exposure time and the percentage of time pH < 4 after baclofen treatment, both not significantly changed compared with the placebo. No significant changes in the percentage of time with pH < 4 were noted for the upright, postprandial, and supine periods compared with placebo. Acid clearance time was not changed significantly after baclofen (*p* = NS).	NR	After Baclofen, the total amount of reflux episodes was significantly reduced (*P* < 0.01). Reflux in the upright position and postprandial reflux were significantly decreased [(*P* < 0.02) and (*P* < 0.02), respectively]. Number of reflux episodes in the supine position was not significantly changed after treatment baclofen. The number of acid reflux episodes was reduced by 36.6%, however this change was not statistically significant compared to placebo. Bolus clearance was not affected by baclofen (*p* = NS)	NR	The total number of reflux episodes extending to the most proximal impedance electrodes was significantly reduced after treatment with baclofen (*P* < 0.05)
Cange et al. (31)	The total amount of belching was significantly decreased (*P* < 0.01), but no effect was observed on heartburn or other symptoms associated with GERD.	A significant reduction was observed in the fraction of time pH < 4 during the first 4 h after dosing with baclofen (*P* = 0.0019), postprandially (*P* = 0.0083) and for the whole 12-h period (*P* < 0.039). However, during the 4–8 and 8–12 h periods, no significant reduction was noted.	NR	After baclofen, a significant reduction in the number of acid reflux episodes, both during the first 4 h after dosing (48%) and during the total 0–12 h recording period (*P* < 0.0001) was recorded.	NR	Baclofen had no significant effect on esophageal clearance.
Cossentino et al. (25)	Baclofen significantly decreased overall symptom score (*P* = 0.004) and symptom scores including belching (*P* = 0.036), regurgitation (*P* = 0.036). No improvement in heartburn score (*P* = 0.186) or chest pain score (*P* = 0.249) and no significant increase in drowsiness after baclofen treatment (*P* = 0.064) was noted.	Significant improvement in 24-h pH score (*P* = 0.020) after treatment with baclofen was noted.	NR	After baclofen, the number of postprandial reflux episodes (*P* = 0.045) and the percent total postprandial reflux decreased significantly (*P* = 0.003). Significant improvements in several reflux parameters including percent upright reflux (*P* = 0.016), percent total reflux (*P* = 0.003), number of reflux episodes (*P* = 0.018) and number of reflux episodes > 5 min (*P* = 0.016) were observed after baclofen treatment. No significant differences in percent supine reflux (*p* = 0.057) or the longest episode of reflux (*p* = 0.063) were observed.	No significant changes in LES pressure after treatment with baclofen (*P* = 0.590) were observed.	NR
Gerson et al. (54)	The mean number of heartburn events associated with acid reflux episodes or non-acid reflux episodes for all dose groups combined was significantly reduced.	NR	NR	There was a statistically significant reduction in reflux episodes during treatment with AP compared with placebo treatment.	NR	NR
Grossi et al. (32)	NR	NR	The number of TLESRs was significantly reduced [basal 57.5; baclofen 41.5 (*P* = 0.01); placebo 55 (*P* = NS)]	NR	A significant increase in the basal LES tone was observed [basal 20.5 mmHg; baclofen: 24.8 mmHg (*P* = 0.02); placebo 21.5 mmHg (*P* = NS)]	The number of swallows was reduced significantly after baclofen compared to the baseline (*P* = 0.02)
van Herwaarden et al. (34)	NR	Baclofen significantly reduced the acid reflux time (*P* = 0.029). In contrast, baclofen had no effect on the mean duration of reflux episodes (*P* = NS).	The incidence of TLESR was significantly reduced after the treatment with baclofen compared to placebo (*P* < 0.0001); However, baclofen had no effect on the percentage of TLESR associated with acid reflux (*P* = NS)	Baclofen significantly reduced the number of reflux episodes (*P* = 0.004)	Baclofen had no significant effect on LES pressure (*P* = NS)	NR
Vela et al. (27)	Treatment with baclofen resulted in a significant reduction in the median number of total (*P* = 0.004), acid-related (*P* = 0.008) and non-acid-related (*P* = 0.04) symptoms.	NR	NR	Baclofen significantly reduced the median number of episodes of acid reflux (*P* = 0.004), re-reflux (*P* = 0.02), non-acid reflux (*P* = 0.003), and all reflux combined (*P* = 0.004)	NR	NR
Scarpellini et al. (33)	Baclofen significantly decreased epigastric burning, retrosternal cramps, and abdominal pain in the preprandial period compared to placebo (*P* < 0.05). In the postprandial period, baclofen significantly decreased belching and epigastric burning compared to placebo (*P* < 0.05).	NR	Baclofen did not change the number of TLESRs preprandially but significantly prevented TLESRs from increasing in postprandial period, resulting in a reduction in the occurrence of postprandial TLESRs after baclofen compared with placebo	NR	After placebo LES pressure reduced from 32.7 ± 6.1 before meal to 24.5 ± 3.1 mm Hg at 60 min postprandially, *P* < 0.05). In contrast, baclofen prevented the postprandial drop in LES pressure (25.4 ± 7.0 preprandially vs. 29.4 ± 4.9 mm Hg at 60, *P* = NS).	The proximal extent of acid pockets detected postprandially was not affected by baclofen or placebo (*P* = NS).
Zhang et al. (11)	No overall significant differences in changes in symptom scores after baclofen administration compared to placebo were noted.	Although reflux episodes were significantly reduced, esophageal acid exposure was not significantly affected by baclofen. The duration of time that esophageal pH was <4 was not changed during baclofen compared to that during placebo (*p* = NS).	The rate of TLESRs per 3 h was significantly reduced (*p* < 0.0002) after baclofen. Baclofen had no effect on the likelihood of reflux occurring during a TLESR. Reflux occurred with 53.8% (40.9–67.3%) of TLESRs during baclofen compared to 41.5% (25.0–75.5%) during placebo.	After baclofen administration, 101 acid reflux episodes happened compared to 174 during placebo. For the postprandial period as a whole, the rate of reflux episodes was reduced by 43% from 7.0 episodes per 3 h to 4.0 episodes per 3 h after baclofen (*p* < 0.02).	LES pressure in fasting basal and first 90 min postprandial after baclofen was similar to placebo; however, basal LES pressure during baclofen was significantly higher in 90–180 min postprandial, compared to placebo (*p* < 0.005), and overall mean postprandial basal LES pressure was significantly higher during baclofen compared to placebo (*p* < 0.02).	Rate of swallowing decreased significantly per 3 h (*p* < 0.05). Fasting or postprandial esophageal peristalsis was not affected by baclofen. Baclofen had no effect on the success rates of both primary and secondary peristalsis and distal esophageal wave amplitude.
Orr et al. (30)	NR	The recumbent acid contact time was decreased by approximately 50% in baclofen compared with placebo, although this deacrease was not statistically significant.	NR	Reflux events occurred during wakefulness and sleep but the number of reflux events were significantly lower during sleep (*P* < 0.05). Baclofen reduced reflux events associated with wakefulness and sleep compared with placebo (*P* < 0.05).	NR	Baclofen significantly improved objective and subjective measures of sleep compared to placebo. Baclofen increased total sleep time (*P* < 0.001) and sleep efficiency (*P* < 0.001), decreased stage 1 sleep (*P* < 0.01) and wake time after sleep onset (*P* < 0.001). Baclofen also significantly improved the subjective number of awakenings (*P* < 0.05) and quality of sleep (*P* < 0.01) compared to placebo. Onset latency, the percent of REM sleep or slow-wave sleep was not affected by baclofen compared to placebo.
Vakil et al. (26)	Change from baseline in weekly heartburn events in baclofen compared to placebo was not statistically significant; however, a significant interaction was noted between prior PPI use and response to baclofen treatment. In the PPI-responsive subgroup, percent reductions from baseline in weekly heartburn events were higher for each baclofen dose vs. placebo (*P* < 0.05) and the percentage of subjects who reported complete resolution of heartburn during week 4 was higher in each baclofen treatment group (21, 28, 30, and 50% for baclofen 20, 40, 60 mg daily, and 30 mg twice daily, respectively) compared to placebo (6%)	NR	NR	NR	NR	NR
Koek et al. (20)	After adding baclofen to the treatment, overall symptom severity was significantly reduced (*p* < 0.01). The severity of heartburn, odynophagia, and choking was significantly reduced (*p* < 0.05) and a borderline reduction in the severity of throatache was noted (*p* = 0.07).	Under combination therapy with omeprazole 20 mg BD and baclofen 20 mg TDS, esophageal acid exposure was unchanged but distal esophageal exposure duodenal reflux was significantly reduced (*p* < 0.05).	NR	The number of acid reflux episodes was unchanged after addition of baclofen. The number of duodenal reflux episodes and the number of duodenal reflux episodes lasting longer than 5 min were significantly reduced. Duodenal reflux exposure during treatment with baclofen was decreased both in the upright and supine positions.	NR	NR
Bajbouj et al. (19)	None of the patients experienced a significant resolution of symptoms after adding baclofen to the treatment.	No significant changes were noted before and after adding baclofen.	NR	No significant changes were noted before and after adding baclofen.	NR	NR

BD, twice daily; GER, gastroesophageal reflux; GERD, gastroesophageal reflux disease; LES, lower esophageal sphincter; NR, not reported; NS, not significant; PPI, proton pump inhibitor; TDS, three times a day; TLESR, transient lower esophageal sphincter relaxation; vs, versus.

##### 3.4.2.2. Outcomes in children

Six studies were included in this category. The mean age of the participants ranged between 7.1 months and 10.0 years.

Four studies reported changes in clinical status. All of them confirmed baclofen’s significant efficacy in the improvement of clinical status by symptom remission, weight gain, or reduction in crying and restlessness.

Three studies evaluated the efficacy of baclofen in children using invasive GI procedures (pH monitoring or esophageal manometry). Omari et al. (35) and Dibner (17) found that children receiving baclofen had considerably lower TLESR and acid GER compared to children receiving a placebo. In Kawai et al.’s (24) study, the total number of acid reflux events was reduced significantly during the postprandial and entire 24-h periods. However, they found no significant changes in total acid exposure time, the percentage of time with esophageal pH < 4 and the duration of the longest acid reflux resulting from baclofen (24) (Table 6).

**TABLE 6:** Outcomes in children.

References	Age (case/control)	Baclofen effect on	
		Clinical status	Others
Dibner et al. (17)	Range: 2.6–17.4 years	NR	Children receiving baclofen had significantly less TLESR and acid GER in the test duration than in the control duration and for the placebo group, no differences were detected. Children receiving baclofen had faster gastric emptying and a higher frequency of normal gastric emptying than those who received the placebo.
Omari et al. (35)	10.0 (9.1/11.0) years	NR	In the control period, the average number of acid GER episodes and the proportion of TLESR-associated acid GER episodes in the placebo group were significantly lower than in the baclofen group. Children receiving baclofen 1 h before the second drink, recorded significantly fewer TLESRs and acid GER episodes during the test in comparison with the control period. For children receiving the placebo, no significant differences between the test and control periods for the frequency of TLESRs and reflux were recorded.
Sobhani Shahmirzadi et al. (21)	6.61 years	85.2% of cases in the baclofen treatment group and 55.2% of cases in the non-baclofen treatment group had moderate to full remission. Weight gain in the baclofen-treated group was significantly higher than in the non-baclofen group (*p* = 0.0001)	NR
Vadlamudi et al. (55)	6.1 years	66% of patients showed a significant reduction in clinical symptoms at their first follow-up visit. Baclofen was stopped in the remaining 34% of patients because of either no response (28%) or adverse events (6%). A total of 27 patients continued treatment and were assessed for long-term response. Of those 81% had a sustained response to baclofen at 12 months, whereas 19% lost response.	NR
Kawai et al. (24)	3 years	The emesis score was significantly decreased (*P* = 0.03)	The incidence of acid refluxes was significantly decreased during the entire 24-h period (*P* = 0.01) and the postprandial period (*P* = 0.049). The number of long acid refluxes (>5 min) was significantly decreased during the 24-h period (*P* = 0.02), but there was no significant difference during the postprandial period (*p* = 0.21). The percentage of total time with esophageal pH < 4.0, the duration of the longest acid reflux, and esophageal acid clearance time had no significant change with therapy either during the 24-h period or during the postprandial period (*p* > 0.05)
Khodadad et al. (29)	7.1 months	The average weight gain of patients was significantly increased (*p* < 0.0001). Crying and instability were significantly decreased (*p* < 0.0001). Vomiting was significantly decreased (*p* < 0.0001). The feeding frequency was significantly increased (*p* < 0.001).	NR

GER, gastroesophageal reflux; NR, not reported; TLESR, transient lower esophageal sphincter relaxation.

##### 3.4.2.3. Outcomes in patients with GERC

In this category, four studies were included. Cough period ranged from 12.6 to 36 months on average. The daytime cough symptom score was greater (3, 4) than the night time cough symptom score (1, 2). Overall, baclofen was effective in treating GERC in 122 of 214 (57.0%) individuals. Outcomes are available in more detail in Table 7.

**TABLE 7:** Outcomes in patients with GERC.

References	Cough duration (months)	SAP for acid reflux (%)	SAP for non-acid reflux (%)	Cough symptom score (daytime/ nighttime)	Demeester score	Baclofen effect on cough
Xu et al. (56)	Patient 1: 42, patient 2: 24, patient 3: 25	Patient 1: 0.0, patient 2: 97.3, patient 3: 84.4	Patient 1: 95.2, patient 2: 0.0, patient 3: 0.0	Patient 1:(3/1), patient 2:(4/2), patient 3:(3/2)	Patient 1: 0.7, patient 2: 168.1, patient 3: 20.2	Cough reduced and waned in all 3 patients (approved for improvement of cough symptom score and cough reflex sensitivity to capsaicin)
Xu et al. (23)	36	73.1	71.2	(3/1)	33.1	The overall therapeutic efficacy of baclofen was 56.3% (9/16). In the remaining 7 patients who withdrew baclofen therapy (*n* = 4) or were resistant to treatment (*n* = 3), the cough was resolved by subsequent therapies of the double dose of omeprazole in 5 patients and a double dose of omeprazole combined with ranitidine in 2 patients.
Xu et al. (22)	NR	75.3	69.1	NR	29.9	Baclofen was effective in cough resolution 66.7% of the patients with refractory GERC who failed to respond to high-dose omeprazole and ranitidine
Zhu et al. (42)	12.6	52.3	78	(3/1)	NR	The overall therapeutic successful rate of baclofen was 52.2% (72/138). For 66 patients who either withdrew from baclofen therapy (*n* = 10) or were unresponsive to baclofen treatment (*n* = 56), the cough was resolved by a double dose of omeprazole in 57 patients or by the consequent therapies combining gabapentin with omeprazole in 9 patients

GERC, gastroesophageal reflux-induced chronic cough; NR, not reported; SAP, symptom associated probability.

##### 3.4.2.4. Outcomes in patients with HH

Three studies were included in this category because they featured a subgroup of patients with HH. Cange et al. (31) reported a significant reduction in acid reflux time and reflux episodes after receiving baclofen, compared to placebo. Beaumont et al. (15) found no significant changes in total acid exposure time and the percentage of time with pH < 4 in patients with HH after administration of baclofen and placebo. However, they observed that baclofen reduced the total number of reflux episodes compared to placebo. In addition, they made a comparison between patients with and without HH and found: (I) patients with a large HH did not show a significantly more proximal reflux compared to patients without HH. No correlation was found between the size of the HH and the proximal extent of the reflux (*r* = 0.1; *P* = 0.75); (II). The total number of reflux episodes of placebo was significantly higher in patients with HH and remained significantly higher compared to in patients without HH (*P* < 0.05). No correlation (*r* = 0.01; *P* = 0.95) was found between the size of the HH and the number of acid reflux episodes (15). In Scarpellini et al.’s (33) study, preprandial LES pressures following baclofen were significantly lower in patients with HH compared to those without HH (*P* < 0.05), and did not change significantly in the postprandial period (Table 8).

**TABLE 8:** Outcomes in patients with HH.

References	Hiatal hernia length	Baclofen effect on		
		Acid reflux time	GER incidence	Others
Beaumont et al. (15)	>3 cm	During baclofen, the total acid exposure time and the percentage of time esophageal pH < 4, both showed no significant difference compared with the placebo (*p* = NS). During baclofen, no significant changes in the percentage of time with pH < 4 were observed for the upright, postprandial and supine period compared with the placebo. Baclofen had no significant effect on acid clearance time.	A significant reduction in the total amount of reflux episodes was recorded (*P* = 0.003, corresponding with a reduction of 43.3%), but the number of acid reflux episodes had no significant reduction by baclofen. Reflux in the upright position was significantly lower (*P* = 0.003), but reflux in the supine position showed no significant reduction by baclofen. Baclofen significantly reduced the amount of mixed (*P* = 0.003) and pure liquid (*P* < 0.02) reflux episodes.	The amount of most proximal reflux episodes was significantly reduced after baclofen (*P* = 0.005, corresponding with a reduction of 57.1%). The proportion of all reflux episodes that reached the most proximal extent was not significantly changed by baclofen (placebo: 21.8%; baclofen: 22.8%)
Cange et al. (31)	NR	In hiatal hernia patients (*n* = 13) a significant reduction was found for the fraction of time pH < 4 during the first 4 h after dosing (*P* = 0.0215) and post-prandially, both during the first and second 4-h periods (*P* < 0.05).	In patients with a verified hiatus hernia (*n* = 13), a significant reduction was found in the number of reflux episodes for the whole registration time (*P* < 0.0002) as well as for each 4-h period (*P* < 0.05).	NR
Scarpellini et al. (33)	>2 cm	NR	NR	Preprandial LES pressures after baclofen were significantly reduced in patients with HH compared with those without HH (*P* < 0.05), and had no significant change in the postprandial period.

GER, gastroesophageal reflux; HH, hiatal hernia; LES, lower esophageal sphincter; NR, not reported.

#### 3.4.3. Feasibility of using baclofen in the treatment of GERD

Table 9 summarizes the evidence from the included studies regarding the feasibility of using baclofen in the treatment of GERD.

**TABLE 9:** Feasibility of using baclofen in the treatment of GERD.

References	Does the study recommend baclofen for GERD management?	Baclofen suggested for (or not suggested for)
Curcic et al. (28)	Yes, reduces the frequency of TLESRs and reflux events after meals and also reduces the esophagogastric insertion angle maintains the intra-abdominal LES segment which may suppress reflux	GERD patients
Abbasinazari et al. (18)	Yes, reduces GERD related symptoms	GERD patients
Ciccaglione et al. (16)	Yes, baclofen in multiple doses reduces gastroesophageal acid reflux for a 24-h period in terms of the number and period of acid exposure in the esophagus, also improves reflux parameters and symptoms related to GER in long term therapy	GERD patients
Beaumont et al. (15)	Yes, reduces the number of reflux episodes	GERD patients with or without hiatal hernia and incomplete response to acid suppression
Cange et al. (31)	Yes, reduces the number of reflux episodes and the fraction of time with esophageal pH < 4	GERD patients
Cossentino et al. (25)	Yes, improves pH parameters and symptoms	GERD patients, may be more effective in patients with predominantly upright reflux and belching
Dibner et al. (17)	Yes, decreases GER by inhibiting TLESRs and accelerating gastric emptying	Children with GERD
Gerson et al. (54)	Yes, reduces reflux episodes and associated heartburn symptoms.	GERD patients
Grossi et al. (32)	Yes, decreases the frequency of TLESRs and elicits a greater basal LES tone	GERD patients
Omari et al. (35)	Yes, reduces GER by inhibiting the triggering of TLESR and accelerating Gastric emptying	Children with GERD
van Herwaarden et al. (34)	May, reduce acid reflux time but couldn’t decrease symptoms. Baclofen decreases post-prandial acid reflux by reducing the incidence of transient lower esophageal sphincter relaxations	GERD patients
Vela et al. (27)	Yes, reduces post-prandial acid and non-acid reflux and their associated symptoms	GERD patients with heartburn as the predominant symptom
Scarpellini et al. (33)	Yes, although baclofen was able to significantly improve upper GI symptoms both preprandially and postprandially, the effect on symptoms does not depend on a change in the extent of the acid pocket	Heartburn-prevalent GERD patients
Zhang et al. (11)	May, baclofen significantly inhibits gastroesophageal reflux episodes by inhibition of TLESRs	GERD patients
Orr et al. (30)	Yes, reduces the number of reflux events during sleep and significantly improves several measures of sleep. Therefore, could be considered as a useful adjunct therapy to PPIs	GERD patients with sleep disturbances or nighttime heartburn resistant to PPI therapy
Sobhani Shahmirzadi et al. (21)	Yes, along with routine gastroesophageal reflux treatments in children can help reduce or improve symptoms of the disease	Children 6 months to 12 years old with GERD
Vakil et al. (26)	Yes, but just if taken with PPI	GERD patients with prior use of PPIs
Vadlamudi et al. (55)	Yes, reduces reflux events by inhibiting TLESRs and can be used as supplemental therapy to PPI	Children with refractory GERD in combination with PPIs
Xu et al. (56)	Yes, decreases cough symptom score and cough reflex sensitivity to capsaicin	GERC patients resistant to PPIs
Xu et al. (23)	May, when a standard therapy for GERC fails, baclofen can at least be considered as a treatment option, even though its therapeutic efficacy is suboptimal	GERC patients unresponsive to standard anti-reflux therapy
Xu et al. (22)	Yes, decreases cough symptom score and cough reflex sensitivity to capsaicin	GERD patients unresponsive to PPI plus H2-blocker
Zhu et al. (42)	No, may not be strong enough to allow a routine clinical use	(Patients with refractory GERC)
Kawai et al. (24)	Yes, Repetitive administration of baclofen reduces the frequency of emesis and the total number of acid refluxes	Neurologically impaired children with GERD
Khodadad et al. (29)	Yes, controls the occurrence of vomiting and reduces instability of infants, and causes gaining weight and improvement of nutrition. It could be used as a replacement for prokinetics to treat GERD.	Infants with GERD
Koek et al. (20)	Yes, improves duodenal reflux and associated reflux symptoms that persist during PPI therapy, inhibits the number of TLESRs, and decreases reflux events and duodenal reflux exposure	GERD patients with reflux that persisted during PPI treatment as add-on therapy
Bajbouj et al. (19)	No, baclofen administration as an add-on therapy showed inconclusive results concerning reflux events.	(GERD patients with persistent GER despite therapy with PPI at the standard dose and double dose)

GER, gastroesophageal reflux; GERC, gastroesophageal reflex-induced chronic cough; GERD, gastroesophageal reflux disease; H2-blocker, histamine-2 blocker; LES, lower esophageal sphincter; PPI, proton pump inhibitor; TLESR, transient lower esophageal sphincter relaxation.

## 4. Discussion

### 4.1. Summary of the main results

We conducted this study to review the effects of baclofen on the treatment of GERD along with its advantages and disadvantages. Most included studies we reviewed diagnosed GERD based on clinical presentation and only a few diagnosed on pH-monitoring results. The results showed that baclofen is a relatively safe choice that may significantly improve reflux symptoms and pH-monitoring and manometry findings, although its effect on pH-monitoring parameters seems less significant than the other parameters. Baclofen showed effective to different degrees in all of four assessed categories (including adults, children, patients with GERC, and patients with HH).

The mechanisms, safety, and efficacy of baclofen in the GERD management will be discussed in the following sections.

### 4.2. Mechanisms

Baclofen is a GABA agonist that works primarily in the spinal cord by binding to GABA_*B*_ receptors and inhibits the release of substance P and excitatory neurotransmitters; so, baclofen alleviates muscle spasms and discomfort (36). Although the spinal cord is the principal site of action for baclofen, its receptors are also found in the brain. Studies suggest that baclofen interacts with serotonin, dopamine, and other neurotransmitters, an off-label treatment for post-traumatic stress disorder and anxiety (37).

However, how does baclofen work to treat gastroesophageal reflux? The molecular and neural mechanisms of action of baclofen in reflux disease are still unclear. The vasovagal reflex relaxes the LES as food enters the stomach, but predisposes to acid and food reflux to occur. As a result, the TLESR is the most likely cause for the gastroesophageal reflux disease. Number of neurotransmitters play a role in this reflex, but GABA_*B*_ receptor agonists have received the most attention for drug intervention. A low basal pressure of the LES is another proposed mechanism for reflux disease (38, 39). All included clinical trials reported the significant effect of baclofen on TLESRs and LES pressure, except for Cossentino et al. (25) and van Herwaarden et al. (34) who found no significant change in LES pressure between the baclofen and placebo groups. They prescribed baclofen for a period of 2 weeks and 1 day, respectively. Their result does not seem to be attributable to the period of baclofen administration, since there are some studies reporting the efficacy of baclofen on LES pressure during the same administration period (11, 28, 32, 33).

There are still many questions about the mechanism of baclofen in GERD. Although the manometry findings confirm the effect of baclofen on the LES, baclofen has been less effective on pH-monitoring findings. Unlike PPIs, baclofen has no known effect on gastric acid secretion, but theoretically, it may reduce acid exposure time secondary to increased LES pressure. However, most of the studies reported no significant improvement in acid exposure time with baclofen. Furthermore, in some of these studies, despite no significant reduction in acid exposure time, GERD symptoms were significantly improved. This can suggest other mechanisms rather than the effect on the LES; for example, baclofen may have a role in suppressing esophageal sensory neurons, as a result, despite the reflux of the acid, patient feels no heartburn. Taken together, it seems that the increased LES pressure cannot be the only mechanism of action of baclofen in GERD; more studies are needed in this field.

### 4.3. Safety and side effects

One of the most critical considerations in any treatment is the patient’s safety. Baclofen’s side effects have caused hesitancies in prescribing for the treatment of GERD.

As previously mentioned, baclofen is a GABA_*B*_ agonist which justifies its side effects by this mechanism. CNS side effects may include dizziness, drowsiness, confusion, sedation, asthenia, and nausea. These side effects are dose-dependent and related to the pharmacologic action of binding to the presynaptic GABA_*B*_ receptors within the brain stem, dorsal horn of the spinal cord, and other CNS parts while reducing the release of excitatory neurotransmitters. Taking oral doses of more than 60 mg per day and severe renal impairment (eGFR less than 30 ml/minute/1.73 m^2^) are the major predictors for CNS side effects. Patients who are concurrently taking other CNS depressants (for example, benzodiazepines or opioids) are more susceptible to these side effects (40, 41).

As expected, these side effects were also observed in the clinical trials. These side effects were well explained both by the agonistic effects of baclofen on GABA_*B*_ receptors in the central and peripheral nervous systems and the normal postprandial symptoms of GERD. The duration of the treatment and other factors may have an impact on its safety. We review that in short -term use the overall adverse effects of baclofen in GERD patients are negligible, yet in long-term use side effects are more prominent. However, Sobhani Shahmirzadi et al. (21) and Khodad et al. (29) administered baclofen for a period of 1 month and 3 months, respectively; and no significant adverse event was observed despite long-term use of baclofen.

Li et al. (12) conducted a meta-analysis of nine RCTs to examine the safety of baclofen in reflux therapy. Baclofen- and placebo-treated participants did not have a statistically significant difference in the frequency of overall adverse events (OR = 1.62; 95% CI: 1.03, 2.54; *P* = 0.04). Neurological/psychiatric symptoms were the most reported side effects. All reported adverse events were mild to moderate in intensity (12).

Some clinicians administer low dose baclofen and increment the dosage as a precaution against baclofen adverse effects. This approach was utilized in six of our trials (15, 16, 19, 20, 25, 42). However, results are inconclusive due to lack of studies comparing high-dose baclofen at the beginning of treatment with the incremental dosing mentioned above in adverse effects.

This review concludes an acceptable safety and tolerability profile for baclofen in GERD, yet Caution should be taken in the long-term use of baclofen, as 20% of long-term users experienced neurological and mental side effects. We noted that some trials did not report side effects, and some had restricted criteria toward side effects; therefore, side effects might not be reported in these studies. Furthermore, the populations of the included studies were heterogeneous. Thus, we recommend that cautions should be considered when administering baclofen to susceptible populations. In general, to limit the risk of baclofen side effects, we recommend: (i) starting with a low dose and gradually increasing it; (ii) prescribing no more than 60 mg of baclofen per day; and (iii) obtaining a comprehensive history of the patient, including comorbidities, medications (particularly CNS depressants) and a previous history of dizziness, somnolence and other baclofen side effects.

### 4.4. Efficacy

In our included trials, the efficacy of baclofen was evaluated through changes in clinical status, acid reflux time, TLESR incidence, GER incidence, LES pressure, and several other factors. Due to heterogeneity of the included studies, a meta-analysis of the data was not possible. Nonetheless, the effects of baclofen on GERD from the results are noticeable. Li et al. (12) conducted a meta-analysis on nine RCTs to determine the efficacy of baclofen on reflux statistically (eight studies administered baclofen in GERD patients and one administered baclofen in normal healthy subjects). The results revealed a statistically significant difference between baclofen-treated and placebo-treated subjects in reduction of GER incidence [standardized mean difference (SMD): −0.65; 95% CI: −0.94, −0.36; *P* = 0.00001], acid reflux time (SMD: −1.14; 95% CI: −1.72, −0.56; *P* = 00001) and TLESR incidence (SMD: −3.56; 95% CI: −4.30, −3.00; *P* < 0.00001) (12).

Baclofen may benefit a diverse population including: adults, infants with refractory regurgitation, neurologically impaired children with GERD, patients with GERC, and patients with hiatal hernia may benefit from baclofen. Of the twenty-six included, only Bajbouj et al. (19) and Zhu et al. (42) advised against using baclofen for the treatment of reflux disease.

The study by Bajbouj et al. (19) involved seven patients with GERD who did not respond to PPI. They added 15 mg of baclofen to the 80 mg of esomeprazole daily, which was increased to 60 mg after 1 month. They maintained this regimen for a period of 2 months. They found no significant change in patients’ clinical status, acid reflux time and GER incidence after 3 months of treatment. Also, two patients discontinued the study because of drowsiness. Therefore, Bajbouj et al. (19) recommended against the use of baclofen in patients who did not respond to PPI (19). However, in all other studies, baclofen was significantly more effective than placebo in treating GERD patients who did not respond to PPI; we cannot independently explain Bajbouj et al.’s (19) study results.

Among PPI-resistant patients, when deciding to prescribe baclofen, how to distinguish responders from non-responders? The study by Zhu et al. (42) included 138 patients with refractory GERC. In contrast to Bajbouj et al.’s (19) study, baclofen was effective in alleviating symptoms, with 72 of 138 (52.2%) GERC patients receiving a successful treatment. Nonetheless, a significant proportion of patients experienced CNS side effects as a result of long-term baclofen use, and the improvement in cough was not satisfactory. So, Zhu et al. (42) recommended against using baclofen because of its unsatisfactory efficacy and side effects. They discovered that LES pressure with a cut-off point of 11 mmHg (with a sensitivity of 83.1% and a specificity of 79.1%) and LES length with a cut-off point of 2.35 cm (with a sensitivity of 81.6% and a specificity of 72.1%) are the independent predictors of baclofen efficacy in reflux disease (42). Although the population of the study was limited to patients with refractory GERC, considering the mechanism of baclofen on GERD, it may be possible to use these cut-off points for all patients with refractory GERD; but more studies are needed to determine these cut-offs. However, using manometry to assess response to baclofen is impractical, as it is used in a limited portion of GERD patients.

Is baclofen a proper choice in patients with GERC? 64% of individuals in this category were the patients of Zhu et al.’s (42) study. The issue that is remarkable about their study is that they prescribed baclofen as monotherapy without PPI. 52.2% of patients were treated with baclofen and 86.3% of baclofen non-responders were treated with double dose of PPI (42). On the other hand, Xu et al.’s (23) reported that adding baclofen to the PPI was effective in cough resolution of 66.7% of patients with GERC who failed to response high-dose PPI and H2blocker. On the whole, the heterogeneity of the studies in this category prevents us from making a correct judgment, and it seems that baclofen lacks the potency for standard clinical use in patients with GERC, apart from the fact that the risk of side effects is also higher in these patients due to the long-term use.

Does HH reduce the efficacy of baclofen during PPI add-on therapy? Beamount et al. (15) studied 27 GERD patients, including 16 patients without HH and 11 without a large HH. The total number of reflux episodes decreased by 36% in patients without HH and 43% in patients with HH, but the number of acid reflux episodes and total acid exposure time did not change. They reported that baclofen may also be effective in patients with a large HH but findings are not satisfactory (15).

Is baclofen effective as a stand-alone treatment for GERD? Twenty-two studies used baclofen as monotherapy and six used it as a combination therapy with PPI. Baclofen improved symptoms in both treatment groups significantly, but due to the heterogeneity of studies, it is impossible to compare these two groups. Despite the fact that baclofen monotherapy is effective in treating GERD, it is not recommended to use it as the first-line treatment without PPI; especially for long-term use, in which case its side effects are more pronounced. We recommend that, if high-dose PPI treatment fails to improve GERD, baclofen can be added to PPI to benefit the synergistic effects. In PPI-resistant patients, baclofen could be used as a replacement for prokinetics such as domperidone; particularly in patients who have heart diseases, as prokinetics prolong the QT interval and increase the risk of torsades de pointes and fatal arrhythmias (43).

Does body posture affect the efficacy of baclofen? Due to the strain of abdominal organs, the LES pressure is significantly higher in the supine position than the upright position and a low basal pressure of the LES is one of the important mechanisms of the GERD (44). Theoretically, baclofen, which increases the pressure of the LES, will be more effective in upright reflux; because of the lower LES pressure in this posture. Two studies evaluated the effect and reported that baclofen significantly decreased reflux episodes only in the upright position (15, 25). Thus, it seems that baclofen is not a good choice in patients with reflux in supine position (e.g., patients with nocturnal reflux). In contrast, Orr et al. (30) evaluated the efficacy of baclofen in reducing reflux during sleep (supine position). They reported that baclofen can not only significantly reduce reflux events during sleep, but also improve objective and subjective measures of sleep (30). So, it seems that the knowledge about the efficacy of baclofen in upright and supine positions are still paradoxical and more studies are needed to provide an answer.

Adding baclofen or suggesting anti-reflux surgery? After inadequate response from PPIs, the management of GERD is complex (45). Spechler et al. (46) compared the efficacy of medical treatment versus surgical treatment for refractory heartburn. 25 patients received baclofen plus omeprazole and 27 patients underwent nissen fundoplication surgery. The treatment success rate with surgery was significantly higher than medical treatment (67% against 28%, *p*-value = 0.007). Although the study evaluated heartburn (which is not specific for GERD, and also is not the only symptom of GERD), baclofen appears to be a less effective alternative for surgery candidates (46).

### 4.5. Other considerations

Baclofen may be more effective in some populations when other comorbidities of patients with GERD are considered. In addition to patients with muscle spasms, baclofen can be a priority in GERD patients with the following disorders:

#### 4.5.1. Alcohol use disorder (AUD)

Alcohol is one of the substances that can relax the LES and exacerbates reflux symptoms. Patients with symptomatic GERD are frequently advised to abstain from alcohol (47). Moreover, studies have shown that 30–80 mg of baclofen per day could be effective in quitting alcohol or preventing relapse: an off-label indication (48). In symptomatic GERD patients with AUD, baclofen not only reduces symptoms by decreasing TLESR episodes but also aids in alcohol cessation as a lifestyle modification.

#### 4.5.2. Non-acid reflux and rumination syndrome

Antacid medications (including histamine-2 blockers and PPIs) are the first-line treatment for GERD. However, in patients with non-acid reflux or rumination syndrome, the antacid approach does not relieve symptoms. In these patients, increasing LES pressure and minimizing TLESR episodes may be the best option (49, 50). Thus, if pH monitoring reveals non-acid reflux, baclofen could be the treatment of choice.

#### 4.5.3. Obesity

Studies have shown that central obesity is associated with symptomatic GERD. The mechanism is thought to be associated with increasing the gastroesophageal pressure gradient and shortening of the lower esophageal sphincter, which baclofen can resolve the latter. Moreover, obese patients are at higher risk of long-standing GERD complications, including erosive esophagitis, Barrett esophagus, and esophageal adenocarcinoma (51). A pilot study shows the positive effects of baclofen on weight reduction in obese patients (52). Thus, in obese GERD patients, baclofen could reduce both the GERD symptoms and body weight, which is one of the risk factors for symptomatic GERD.

### 4.6. Finally, when and how?

**FIGURE 2:**
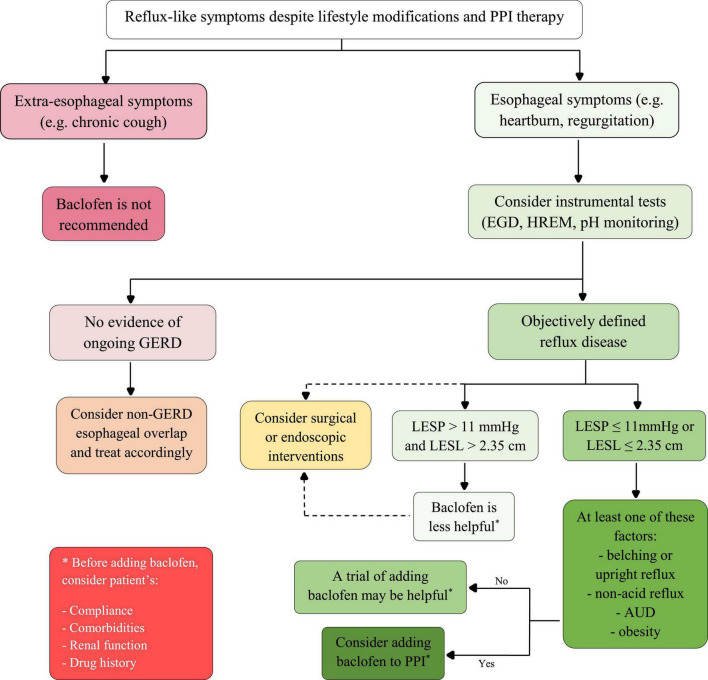
Suggested algorithm for administration of baclofen for refractory reflux disease. AUD, alcohol use disorder; EGD, esophagogastroduodenoscopy; GERD, gastroesophageal reflux disease; HREM, high resolution esophageal manometry; LESL, lower esophageal sphincter length; LESP, lower esophageal sphincter pressure; PPI, proton pump inhibitor. *Means to pay attention to the issues that are listed in red rectangle.

Management of refractory GERD can be very challenging. In PPI non-responders, the American College of Gastroenterology recommends against adding medications other than PPI to the regiment (53); Sometimes it is inevitably necessary to add other medications. Although trials confirm the efficacy of baclofen as a stand-alone treatment for GERD, we do not recommend it as a mono-therapy. In PPI-resistant patients, a trial of adding baclofen to the PPI may be helpful under special circumstances. This can help reduce symptoms (regurgitation, heartburn, and belching) and may decrease the dose of PPI. We recommend against using baclofen in patients with extra-esophageal reflux symptoms (e.g., GERC); as the efficacy of baclofen is low and the side effects are more frequent and severe. Also, we recommend against using baclofen for maintenance therapy or long-term use. In patients with symptomatic reflux who are candidates for anti-reflux surgery but refuse, baclofen can be a modest alternative. However, these patients may experience more side effects of baclofen as well. To reduce side effects, starting baclofen with a dose of 5–10 mg and incrementing to a maximum dose of 60 mg is recommended. Symptomatic GERD patients (especially those with belching or upright reflux) with an AUD, non-acid reflux, or obesity may benefit more from baclofen. An esophageal manometry (measures LES pressure and length) can help selecting refractory GERD patients who may respond appropriately to baclofen. Suggested algorithm for administration of baclofen for refractory reflux disease is illustrated in Figure 2.

### 4.7. Limitations

Some limitations of this study should be taken into consideration. First, most of the studies diagnosed GERD (before and after intervention) by symptoms (not pH-monitoring) what is uncertain. Second, the relatively small number of trials in some outcome categories. This may diminish the persuasiveness of the conclusions. Third, the potential influence of the preexisting conditions and the severity of the reflux disease could not be investigated because of the limited information obtained from the reviewed articles. Fourth, as with any systematic review, limitations associated with potential publication bias should be considered. Furthermore, trials’ variability, different patients’ characteristics, and a wide range of outcome measures were other limitations.

## 5. Conclusion

The present study, to the best of our knowledge, is the first study that systematically addresses various aspects of baclofen administration in the spectrum of GERD patients. A trial of adding baclofen to the PPI may be helpful in PPI-resistant patients. Baclofen therapies may be more beneficial for symptomatic GERD patients who suffer AUD, non-acid reflux, or obesity. To reduce the side effects, we recommend starting baclofen with a low dose and increasing it gradually, avoiding prescribing more than 60 mg of baclofen per day, and paying close attention to the patients’ history.

## Data availability statement

The original contributions presented in this study are included in this article/Supplementary material, further inquiries can be directed to the corresponding authors.

## Author contributions

EA and MAbde designed the study. EA, SK, AA, and NT performed the search, study selection, and data extraction. EA and SK wrote the first draft of the manuscript. EA, MAbdi, DA, and MAbde revised the manuscript. All authors contributed to the article and approved the submitted version.
