# Supplementary material for: Baclofen as a therapeutic option for gastroesophageal reflux disease: A systematic review of clinical trials
Source: Front Med (Lausanne). 2023 Feb 17;10:997440. doi: 10.3389/fmed.2023.997440 (PMC9981648; doi:10.3389/fmed.2023.997440)
Supplement: Supplementary file 1 [file Table_1.pdf]

Supplementary Table 1. Papers excluded in full-text screening

Reason for exclusion		Reference
**Baclofen in wrong population (n=6):**	Healthy persons (n=3)	(1-3)
	Rumination syndrome (n=2)	(4, 5)
	Binge eating disorder (n=1)	(6)
**Not focus on baclofen (n=2)**		(7, 8)
**Review articles (n=1)**		(9)
**Full-text not found (n=3)**		(10-12)

1. Beaumont H, Smout A, Aanen M, Rydholm H, Lei A, Lehmann A, et al. The GABA(B) receptor agonist AZD9343 inhibits transient lower oesophageal sphincter relaxations and acid reflux in healthy volunteers: a phase I study. Aliment Pharmacol Ther. 2009;30(9):937-46.
2. Boeckxstaens GE, Rydholm H, Lei A, Adler J, Ruth M. Effect of lesogaberan, a novel GABA(B)-receptor agonist, on transient lower oesophageal sphincter relaxations in male subjects. Aliment Pharmacol Ther. 2010;31(11):1208-17.
3. Lidums I, Lehmann A, Checklin H, Dent J, Holloway RH. Control of transient lower esophageal sphincter relaxations and reflux by the GABA(B) agonist baclofen in normal subjects. Gastroenterology. 2000;118(1):7-13.
4. Blondeau K, Boecxstaens V, Rommel N, Farré R, Depeyper S, Holvoet L, et al. Baclofen improves symptoms and reduces postprandial flow events in patients with rumination and supragastric belching. Clin Gastroenterol Hepatol. 2012;10(4):379-84.
5. Pauwels A, Broers C, Van Houtte B, Rommel N, Vanuytsel T, Tack J. A Randomized Double-Blind, Placebo-Controlled, Cross-Over Study Using Baclofen in the Treatment of Rumination Syndrome. Am J Gastroenterol. 2018;113(1):97-104.
6. De Beaurepaire R, Joussaume B, Rapp A, Jaury P. Treatment of binge eating disorder with high-dose baclofen: A case series. Journal of Clinical Psychopharmacology. 2015;35(3):357-9.
7. Dong R, Xu X, Yu L, Ding H, Pan J, Yu Y, et al. Randomised clinical trial: gabapentin vs baclofen in the treatment of suspected refractory gastro-oesophageal reflux-induced chronic cough. Aliment Pharmacol Ther. 2019;49(6):714-22.
8. Yu Y, Wen S, Wang S, Shi C, Ding H, Qiu Z, et al. Reflux characteristics in patients with gastroesophageal reflux-related chronic cough complicated by laryngopharyngeal reflux. Ann Transl Med. 2019;7(20):529.
9. Blondeau K. Treatment of gastro-esophageal reflux disease: The new kids to block: VIEWPOINT. Neurogastroenterology and Motility. 2010;22(8):836-40.
10. Xu X, Yu L, Chen Q, Shi C, Lv H, Qiu Z, et al. Association of esophageal dysfunction with therapeutic efficacy of baclofen in patients with refractory gastroesophageal reflux-induced chronic cough. Eur Respiratory Soc; 2017.
11. Wu D, Huang Z, Chen S. Efficacy of baclofen combined with esomeprazole and mosapride on refractory gastroesophageal reflux disease. Chinese Journal of Gastroenterology. 2014;19(12):725-9.
12. Chen C-L, Yi C-H, Lei W-Y, Hung J-S, Liu T-T, Wong M-W. Tu1600-Gaba-B Agonist Baclofen Inhibits Acid-Induced Excitation of Secondary Peristalsis but not Heartburn Sensation. Gastroenterology. 2018;154(6):S-963.
