# Supplementary material for: Baclofen as a therapeutic option for gastroesophageal reflux disease: A systematic review of clinical trials
Source: Front Med (Lausanne). 2023 Feb 17;10:997440. doi: 10.3389/fmed.2023.997440 (PMC9981648; doi:10.3389/fmed.2023.997440)
Supplement: Supplementary file 2 [file Table_2.pdf]

Supplementary Table 2. Quality assessment of controlled intervention studies

First author	1. Was the study described as randomized, a randomized trial, a randomized clinical trial, or an RCT?	2. Was the method of randomization adequate (i.e., use of randomly generated assignment)?	3. Was the treatment allocation concealed (so that assignments could Not be predicted)?	4. Were study participants and providers blinded to treatment group assignment?	5. Were the people assessing the outcomes blinded to the participants' group assignments?	6. Were the groups similar at baseline on important characteristics that could affect outcomes (e.g., demographics, risk factors, co-morbid conditions)?	7. Was the overall drop-out rate from the study at endpoint 20% or lower of the number allocated to treatment?	8. Was the differential drop-out rate (between treatment groups) at endpoint 15 percentage points or lower?	9. Was there high adherence to the intervention protocols for each treatment group?	10. Were other interventions avoided or similar in the groups (e.g., similar background treatments)?	11. Were outcomes assessed using valid and reliable measures, implemented consistently across all study participants?	12. Did the authors report that the sample size was sufficiently large to be able to detect a difference in the main outcome between groups with at least 80% power?	13. Were outcomes reported or subgroups analyzed prespecified (i.e., identified before analyses were conducted)?	14. Were all randomized participants analyzed in the group to which they were originally assigned, i.e., did they use an intention-to-treat analysis?
**Curcic et al.** [1]	Yes	Yes	Yes	Yes	Yes	Yes	Yes	Yes	Yes	Yes	Yes	No	Yes	Yes
**Abbasinazari et al.** [2]	Yes	Yes	Yes	Yes	Yes	Yes	Yes	Yes	Yes	Yes	Yes	No	Yes	Yes
**Ciccaglione et al.** [3]	Yes	Yes	Yes	Yes	Yes	NR	Yes	Yes	Yes	Yes	Yes	No	Yes	Yes
**Beaumont et al.** [4]	Yes	Yes	Yes	Yes	Yes	Yes	Yes	Yes	Yes	Yes	Yes	No	Yes	Yes
**Cange et al.** [5]	Yes	Yes	Yes	Yes	Yes	Yes	Yes	Yes	Yes	Yes	Yes	No	Yes	Yes
**Cossentino et al.** [6]	Yes	Yes	Yes	Yes	Yes	Yes	No	No	Yes	Yes	Yes	Yes	Yes	Yes
**Dibner et al.** [7]	Yes	NR	Yes	Yes	Yes	NR	NR	NR	NR	Yes	Yes	No	Yes	NR
**Gerson et al.** [8]	Yes	Yes	Yes	Yes	Yes	Yes	Yes	Yes	Yes	Yes	Yes	Yes	Yes	Yes
**Grossi et al.** [9]	Yes	Yes	Yes	Yes	Yes	Yes	Yes	Yes	Yes	Yes	Yes	No	Yes	Yes
**Omari et al.** [10]	Yes	Yes	Yes	Yes	Yes	Yes	Yes	Yes	Yes	Yes	Yes	No	Yes	Yes
**Van Herwaarden et al.** [11]	Yes	Yes	Yes	Yes	Yes	Yes	Yes	Yes	Yes	Yes	Yes	No	Yes	Yes
**Vela et al.** [12]	Yes	No	Yes	NR	NR	Yes	Yes	Yes	Yes	Yes	Yes	No	Yes	Yes
**Scarpellini et al.** [13]	Yes	NR	Yes	Yes	Yes	No	Yes	Yes	Yes	Yes	Yes	No	Yes	Yes
**Zhang et al.** [14]	Yes	Yes	Yes	NR	NR	Yes	Yes	Yes	Yes	Yes	Yes	No	Yes	Yes
**Orr et al.** [15]	Yes	Yes	Yes	Yes	Yes	Yes	Yes	Yes	Yes	Yes	Yes	No	Yes	Yes

**Sobhani Shahmirzadi et al.**[16]	Yes	Yes	Yes	Yes	No	Yes	Yes	Yes	Yes	Yes	Yes	No	Yes	Yes
**Vakil et al.**[17]	Yes	Yes	Yes	Yes	Yes	Yes	Yes	Yes	Yes	Yes	Yes	No	Yes	Yes

Abbreviations: NR: not reported

1. Curcic J, Schwizer A, Kaufman E, Forras-Kaufman Z, Banerjee S, Pal A, et al. Effects of baclofen on the functional anatomy of the oesophago-gastric junction and proximal stomach in healthy volunteers and patients with GERD assessed by magnetic resonance imaging and high-resolution manometry: A randomised controlled double-blind study. Alimentary Pharmacology and Therapeutics. 2014;40(10):1230-40.
2. Abbasinazari M, Panahi Y, Mortazavi SA, Fahimi F, Valizadegan G, Mohtashami R, et al. Effect of a Combination of Omeprazole Plus Sustained Release Baclofen Versus Omeprazole Alone on Symptoms of Patients with Gastroesophageal Reflux Disease (GERD). Iran J Pharm Res. 2014;13(4):1221-6.
3. Ciccaglione A, Marzio L. Effect of acute and chronic administration of the GABAB agonist baclofen on 24 hour pH metry and symptoms in control subjects and in patients with gastro-oesophageal reflux disease. Gut. 2003;52(4):464-70.
4. Beaumont H, Boeckxstaens GEE. Does the presence of a hiatal hernia affect the efficacy of the reflux inhibitor baclofen during add-on therapy. American Journal of Gastroenterology. 2009;104(7):1764-71.
5. Cange L, Johnsson E, Rydholm H, Lehmann A, Finizia C, Lundell L, et al. Baclofen-mediated gastro-oesophageal acid reflux control in patients with established reflux disease. Aliment Pharmacol Ther. 2002;16(5):869-73.
6. Cossentino MJ, Mann K, Armbruster SP, Lake JM, Maydonovitch C, Wong RK. Randomised clinical trial: the effect of baclofen in patients with gastro-oesophageal reflux--a randomised prospective study. Aliment Pharmacol Ther. 2012;35(9):1036-44.
7. Dibner LBW. A pharmacological option for the treatment of GERD in infants Effect of Baclofen on esophagogastric motility and gastroesophageal reflux in children with gastroesophageal reflux disease: a randomized controlled trial. Revista de Gastroenterología de México. 2006;71(4):544-5.
8. Gerson LB, Huff FJ, Hila A, Hirota WK, Reilley S, Agrawal A, et al. Arbaclofen placarbil decreases postprandial reflux in patients with gastroesophageal reflux disease. Am J Gastroenterol. 2010;105(6):1266-75.
9. Grossi L, Spezzaferro M, Sacco LF, Marzio L. Effect of baclofen on oesophageal motility and transient lower oesophageal sphincter relaxations in GORD patients: a 48-h manometric study. Neurogastroenterol Motil. 2008;20(7):760-6.
10. Omari TI, Benninga MA, Sansom L, Butler RN, Dent J, Davidson GP. Effect of baclofen on esophagogastric motility and gastroesophageal reflux in children with gastroesophageal reflux disease: a randomized controlled trial. J Pediatr. 2006;149(4):468-74.
11. van Herwaarden MA, Samsom M, Rydholm H, Smout AJ. The effect of baclofen on gastro-oesophageal reflux, lower oesophageal sphincter function and reflux symptoms in patients with reflux disease. Aliment Pharmacol Ther. 2002;16(9):1655-62.
12. Vela MF, Tutuian R, Katz PO, Castell DO. Baclofen decreases acid and non-acid post-prandial gastro-oesophageal reflux measured by combined multichannel intraluminal impedance and pH. Aliment Pharmacol Ther. 2003;17(2):243-51.
13. Scarpellini E, Boecxstaens V, Broers C, Vos R, Pauwels A, Tack J. Effect of baclofen on gastric acid pocket in subjects with gastroesophageal reflux disease symptoms. Dis Esophagus. 2016;29(8):1054-63.
14. Zhang Q, Lehmann A, Rigda R, Dent J, Holloway RH. Control of transient lower oesophageal sphincter relaxations and reflux by the GABA(B) agonist baclofen in patients with gastro-oesophageal reflux disease. Gut. 2002;50(1):19-24.
15. Orr WC, Goodrich S, Wright S, Shepherd K, Mellow M. The effect of baclofen on nocturnal gastroesophageal reflux and measures of sleep quality: a randomized, cross-over trial. Neurogastroenterol Motil. 2012;24(6):553-9, e253.
16. Sobhani Shahmirzadi M, Barati L, Ebraimi M, Shiroodbakhshi K. The Efficacy of Baclofen to Treat Gastroesophageal Reflux disease in Children Aged 6 Months to 12 Years: A Clinical Trial Study. International Journal of Pediatrics. 2020;8(5):11287-96.
17. Vakil NB, Huff FJ, Bian A, Jones DS, Stamler D. Arbaclofen placarbil in GERD: a randomized, double-blind, placebo-controlled study. Am J Gastroenterol. 2011;106(8):1427-38.
