# Supplementary material for: Baclofen as a therapeutic option for gastroesophageal reflux disease: A systematic review of clinical trials
Source: Front Med (Lausanne). 2023 Feb 17;10:997440. doi: 10.3389/fmed.2023.997440 (PMC9981648; doi:10.3389/fmed.2023.997440)
Supplement: Supplementary file 3 [file Table_3.pdf]

Supplementary Table 3. Quality assessment of single-arm clinical trial studies

Reference	1. Was the study question or objective clearly stated?	2. Were eligibility/selection criteria for the study population prespecified and clearly described?	3. Were the participants in the study representative of those who would be eligible for the test/service/intervention in the general or clinical population of interest?	4. Were all eligible participants that met the prespecified entry criteria enrolled?	5. Was the sample size sufficiently large to provide confidence in the findings?	6. Was the test/service/intervention clearly described and delivered consistently across the study population?	7. Were the outcome measures prespecified, clearly defined, valid, reliable, and assessed consistently across all study participants?	8. Were the people assessing the outcomes blinded to the participants' exposures/interventions?	9. Was the loss to follow-up after baseline 20% or less? Were those lost to follow-up accounted for in the analysis?	10. Did the statistical methods examine changes in outcome measures from before to after the intervention? Were statistical tests done that provided p values for the pre-to-post changes?	11. Were outcome measures of interest taken multiple times before the intervention and multiple times after the intervention (i.e., did they use an interrupted time-series design)?	12. If the intervention was conducted at a group level (e.g., a whole hospital, a community, etc.) did the statistical analysis take into account the use of individual-level data to determine effects at the group level?
**Vadlamudi et al.[1]**	Yes	Yes	Yes	Yes	Yes	Yes	Yes	NR	Yes	Yes	Yes	Yes
**Xu et al.[2]**	Yes	Yes	Yes	Yes	Yes	Yes	Yes	No	Yes	Yes	Yes	Yes
**Xu et al.[3]**	Yes	Yes	Yes	Yes	Yes	Yes	Yes	No	Yes	Yes	Yes	Yes
**Xu et al.[4]**	Yes	Yes	Yes	Yes	Yes	Yes	Yes	No	Yes	Yes	Yes	Yes
**Zhu et al.[5]**	Yes	Yes	Yes	Yes	Yes	Yes	Yes	No	Yes	Yes	Yes	Yes
**Kawai et al.[6]**	Yes	Yes	Yes	Yes	Yes	Yes	Yes	NR	Yes	Yes	Yes	Yes
**Khodadad et al.[7]**	Yes	Yes	Yes	Yes	Yes	Yes	Yes	NR	Yes	Yes	NR	Yes
**Koek et al.[8]**	Yes	Yes	Yes	Yes	Yes	Yes	Yes	NR	Yes	Yes	NR	Yes
**Bajbouj et al.[9]**	Yes	Yes	Yes	Yes	Yes	Yes	Yes	No	No	Yes	NR	Yes

Abbreviations: NR: not reported

1. Vadlamudi NB, Hitch MC, Dimmitt RA, Thame KA. Baclofen for the treatment of pediatric GERD. J Pediatr Gastroenterol Nutr. 2013;57(6):808-12.
2. Xu X, Chen Q, Liang S, Lü H, Qiu Z. Successful resolution of refractory chronic cough induced by gastroesophageal reflux with treatment of baclofen. Cough. 2012;8(1):8.
3. Xu XH, Yang ZM, Chen Q, Yu L, Liang SW, Lv HJ, et al. Therapeutic efficacy of baclofen in refractory gastroesophageal reflux-induced chronic cough. World J Gastroenterol. 2013;19(27):4386-92.
4. Xu X, Lv H, Yu L, Chen Q, Liang S, Qiu Z. A stepwise protocol for the treatment of refractory gastroesophageal reflux-induced chronic cough. J Thorac Dis. 2016;8(1):178-85.
5. Zhu Y, Xu X, Zhang M, Si F, Sun H, Yu L, et al. Pressure and length of the lower esophageal sphincter as predictive indicators of therapeutic efficacy of baclofen for refractory gastroesophageal reflux-induced chronic cough. Respir Med. 2021;183:106439.
6. Kawai M, Kawahara H, Hirayama S, Yoshimura N, Ida S. Effect of baclofen on emesis and 24-hour esophageal pH in neurologically impaired children with gastroesophageal reflux disease. J Pediatr Gastroenterol Nutr. 2004;38(3):317-23.
7. Khodadad A, Sani MN, Nemat-Khorasani E, Mansouri F. The effect of baclofen on treatment of infancy gastro-esophageal reflux disorder. Iranian Journal of Pediatrics. 2008;18(SUPPL. 1):15-20.
8. Koek GH, Sifrim D, Lerut T, Janssens J, Tack J. Effect of the GABA(B) agonist baclofen in patients with symptoms and duodeno-gastro-oesophageal reflux refractory to proton pump inhibitors. Gut. 2003;52(10):1397-402.
9. Bajbouj M, Becker V, Phillip V, Wilhelm D, Schmid RM, Meining A. High-dose esomeprazole for treatment of symptomatic refractory gastroesophageal reflux disease -A prospective pH-metry/impedance-controlled study. Digestion. 2009;80(2):112-8.
